# Encephalitis due to miliary tuberculosis in a patient with human immunodeficiency virus: A case report

**DOI:** 10.1016/j.jctube.2021.100230

**Published:** 2021-03-19

**Authors:** Rizaldy Taslim Pinzon, Vincent Ongko Wijaya, Dessy Paramitha

**Affiliations:** a Duta Wacana Christian University School of Medicine, Yogyakarta, Indonesia; b Bethesda Hospital, Yogyakarta, Indonesia

**Keywords:** Miliary tuberculosis, Encephalitis, Human immunodeficiency virus

## Abstract

**Background:**

Miliary tuberculosis (TB) is a form of extrapulmonary tuberculosis due to hematogenous dissemination and occurs more frequently in immunocompromised patients. Clinical manifestations are non-specific and varied with the related organ, including central nervous system involvement. We report the first case of encephalitis associated with miliary TB in human immunodeficiency virus (HIV)-the infected patient.

**Case presentation:**

A 39-year-old male presented with severe headache, numbness in the left side of the body, and partial seizure for two weeks. Previously, the patient complaining of several weeks of cough with generalized weakness. Physical examination showed progressive left-sided weakness and numbness. Chest radiograph showed uniform-sized small nodules randomly distributed diffusely throughout the lungs. Plain computer tomography (CT) brain imaging showed hypodensity in the right parietal region. Laboratory findings showed positive for the HIV antibody test, CD4 counts were 84 cells/μL and acid-fast bacilli from sputum. He was administered empirical anti-TB treatment and was discharged without any complications on day 10.

**Conclusion:**

This is a rare cause of encephalitis due to miliary TB infection in HIV patients. Even though central nervous system involvement is rare in miliary TB infection, physicians should be aware of atypical features of the disease and comorbidity that may predispose this infection.

## 1 Introduction

Tuberculosis (TB) is one of the commonest and important opportunistic infections in patients with human immunodeficiency virus (HIV) infection [1], [2]. The data showed as much as 13% of TB cases occurring among patients with HIV infection [3], [4]. From the total number of occurrence, data showed that 1% of the TB patients had central nervous system (CNS) involvement that related to a high mortality rate and permanent neurological sequelae [5], [6].

Encephalitis is one of the rarest CNS involvement of miliary tuberculosis, particularly in immunocompromised patients [7]. To this day, only a few references have reported the involvement of miliary tuberculosis in the CNS, even though the early treatment of CNS infection by TB can tremendously improve the patient condition [8]. Thus we reported a case of encephalitis as a result of miliary tuberculosis infection in a patient with HIV.

## 2 Case presentation

A 39-year-old male was referred to our hospital's neurology department because of severe headache and numbness in the left side of his body with a partial seizure that lasts 1–2 min for 3–4 times a day. The complaint started two weeks before hospital admission and progressively worsened. He had been healthy until two months before. Recently, the patient presents complaining of several weeks of cough with generalized weakness and poor oral intake. He was a non-smoker and denied using illicit drugs or another relevant medical history. He had no family history of TB and denied contact with a person with a TB infection.

On admission, he appeared well: bodyweight, 52 kg; height, 162 cm; temperature, 36.5 °C; blood pressure, 120/80 mmHg; pulse, 72 beats/min; respirations rate, 24/min; oxygen saturation, 98% while breathing ambient air. The neurology examination showed a hemisensory syndrome and 4/5 as the muscle strength score on the upper and lower extremity, respectively, in the left side of the body. All of the cranial nerve examination results were normal.

**Fig. 1:**
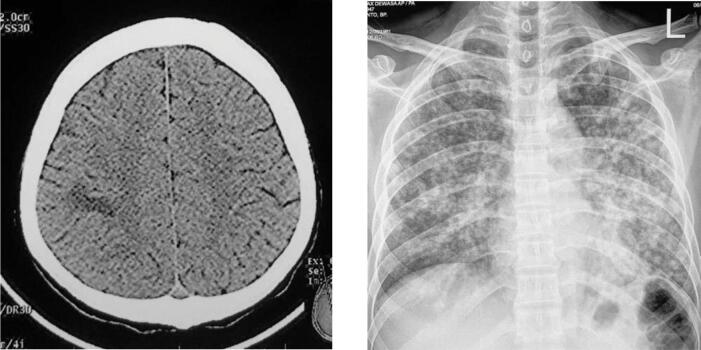
The CT Scan with hypodense lesion and the chest X-ray showed multiple uniform nodule in both lungs.

A computed tomography (CT) scan of the head showed a hypodense lesion in the right parietal lobe. Chest radiograph showed uniform-sized small nodules randomly distributed diffusely throughout the lungs (Fig. 1). Laboratory results found HIV antibody test was positive using ELISA (enzyme-linked immunosorbent assay), and acid-fast bacilli from sputum produced positive results. His CD4 count was 84 cells/μL. We, therefore, diagnosed the patient with miliary TB.

The patient was treated daily with three tablets of a fixed-dose combination of antituberculous treatment consisted of isoniazid (225 mg), rifampicin (450 mg), ethambutol (825 mg), and pyrazinamide (1200 mg), and also streptomycin injection 15 mg/kg. He also received intravenous dexamethasone 0.4 mg/kg per day and mannitol and then tapered-off. Valproic acid was also given for his focal seizure. The symptoms began to improve within three days after he received the anti-TB agents, and he was discharged without any complications on day 10. We postponed the ARV (antiretroviral) therapy within two weeks after starting the Anti-TB medication.

## 3 Discussion and conclusion

It is estimated that miliary TB presents for about less than 2% of total TB cases in immunocompetent patients such as HIV co-infection [7] There is a similar case about a miliary TB patient with headache and cognitive disturbance due to brain tuberculomas reported by Akriditis et al. [9] Another report from Portugal found persistent headache and sleep disturbance that associated with cerebral involvement of miliary TB [10].

The manifestations such as hemiparesis, numbness, focal seizure were atypical features of encephalitis that may have confounded early diagnosis. In the later stage of the disease, encephalitis may be associated with findings such as altered mental status, confusion, or severe headache [8]. In this patient, the CD4 counts less than normal. This immunocompetent state may precipitate the bacterial spread to other organs. The clinical presentation of miliary TB in early HIV infection (CD4 cell counts >200 cells/μL) is similar to that observed in immunocompetent individuals. With the progression of immunosuppression in late, advanced HIV infection (CD4 cell counts <200 cells/μL), disseminated and miliary TB are seen more often [11], [12], [13].

During the progression of the disease, *M. tuberculosis* may disseminate from the lungs to local lymph nodes and bloodstream, spread throughout the systemic circulatory system. The CNS is protected from the systemic circulatory system by the blood–brain barrier (BBB). However, there are several microorganism pathogens capable of passing the BBB and causing subsequent inflammation in brain parenchyma. The well-known theory is rich postulated, termed as “Rich foci,” develop around bacteria deposited in the brain layer and parenchyma during the initial bacteremic phase. Later on, the rupture of these foci led to the dissemination of the bacilli into the subarachnoid space and spread, causing diffuse or focal inflammation in the brain meninges or extensively to its parenchyma [14].

In conclusion, physicians should be aware of comorbidities, particularly infection by HIV is responsible for the etiology of this disease. Early recognition and management of this disorder are important to prevent patient worsening and jeopardize the outcome. Early treatment with antituberculosis therapy (ATT) has been proven to avoid unwanted complications in extrapulmonary miliary TB [7]. Therefore, the reporting of this patient is essential to highlight the atypical features of miliary brain tuberculosis and initiate appropriate care.

## Ethical statement

This study requires no approval from the ethical committee due to the nature of this case report. However, this study has obtained permission from the Bethesda Hospital Research and Development Department. In accordance to Declaration of Helsinki, patient identity was hidden.
